# Poster Session I - A184 HEAT SHOCK PROTEIN 60 OVEREXPRESSION ATTENUATES HEPATIC INFLAMMATION AND FIBROSIS IN NON-ALCOHOLIC STEATOHEPATITIS VIA REGULATION OF MITOCHONDRIAL STRESS–RELATED GENES

**DOI:** 10.1093/jcag/gwaf042.184

**Published:** 2026-02-13

**Authors:** Y Huang

**Affiliations:** Kaohsiung Chang Gung Memorial Hospital Department of Pediatric Internal Medicine, Kaohsiung, Kaohsiung City, Taiwan

## Abstract

**Background:**

Non-alcoholic steatohepatitis (NASH), a severe form of fatty liver disease, results from mitochondrial dysfunction and oxidative stress. Heat shock protein 60 (HSP60) maintains mitochondrial integrity and inhibits inflammatory signaling.

**Aims:**

We explore the role of HSP60 in NASH, including its regulation of mitochondrial health and inflammatory signaling, and examine whether altering HSP60 can reduce steatohepatitis.

**Methods:**

A transgenic mouse model overexpressing HSP60 was fed a methionine- and choline-deficient (MCD) diet for four weeks to induce NASH. Liver histology, RNA sequencing, and qPCR validation were employed to assess hepatic steatosis, fibrosis, and alterations in gene expression in wild-type (WT) and HSP60 transgenic mice fed normal and MCD diets.

**Results:**

HSP60-overexpressing mice exhibited reduced hepatic lipid accumulation and fibrosis compared to WT-MCD mice. Histological and molecular analyses also confirmed decreased hepatic oxidative stress, as shown by the 8-OHdG assay, and reduced inflammatory markers, IL-6 and MCP-1, in HSP60 transgenic mice. RNA-seq revealed 2,709 genes altered in WT-MCD mice, including 1,880 that were upregulated. Ten genes showed opposite regulation in HSP60 transgenics, with five—Dusp1, Parp16, Tczm, Map3k13, and Slc13a3—confirmed by quantitative PCR (qPCR). These genes are associated with inflammation, Wnt/β-catenin signaling, ER stress, and oxidative stress.

**Conclusions:**

HSP60 overexpression protects against diet-induced NASH by reducing oxidative stress and inflammation through regulation of mitochondrial and stress-related pathways, highlighting HSP60 as a potential therapeutic target for metabolic liver disease.

**Funding Agencies:**

114-2314-B-182A-043

